# A Randomized Double‐Blind, Placebo‐Controlled Trial of Artesunate and Curcumin in Patients With Crohn's Disease: A Pilot Study

**DOI:** 10.1002/jgh3.70211

**Published:** 2025-06-29

**Authors:** Devinder Kumar, Uday C Ghoshal, Vamika Mansi Saigal, Moni Chaudhary, Shikha Sahu, Vishruti Pandya, Ujjala Ghoshal

**Affiliations:** ^1^ St George's University of London London UK; ^2^ Department of Surgery Apollo Hospital Ahmedabad India; ^3^ Bowel Disease UK London UK; ^4^ Departments of Gastroenterology and Microbiology Sanjay Gandhi Postgraduate Institute of Medical Sciences Lucknow India; ^5^ Division of Preventive Medicine University of Alabama Birmingham Alabama USA

**Keywords:** Artesunate, azathioprine, Crohn's disease, curcumin

## Abstract

**Aim:**

Less than half of all patients with Crohn's disease remain in remission with long‐term use of azathioprine. Our aim was to assess the efficacy of Artesunate and Curcumin in maintaining remission in patients with Crohn's disease, who had ongoing evidence of disease activity despite taking azathioprine.

**Methods:**

Patients with Crohn's disease being treated with azathioprine for at least 3 months but still had mild to moderate Crohn's disease (CDAI 150–450) were included. Patients were randomized into four blocks of 10 patients each in a 2 × 2 factorial design to receive artesunate 200 mg PO daily for 2 weeks and/or curcumin 200 mg PO daily for 3 months or placebo. Harvey–Bradshaw Index, CDAI, and fecal calprotectin were measured at baseline, 1 week, 1 month, 3 months, and 6 months.

**Results:**

Forty patients were recruited and randomized into the study. Both Artesunate and Curcumin were well tolerated with no adverse effects. The Harvey–Bradshaw Index statistically differed across the treatment groups at 6 months (*p* = 0.047), there were no significant group differences in the post hoc pairwise analysis. The differences in CDAI from baseline to 6 months were statistically significant in Artesunate + Curcumin (*p* = 0.0098) and Curcumin + Placebo (*p* = 0.0431) groups. Similarly, statistically significant differences were observed between Baseline and 6 months for the Harvey–Bradshaw Index in the Artesunate + Curcumin (*p* = 0.0070) and Curcumin + Placebo (*p* = 0.0138) groups.

**Conclusion:**

A combination of artesunate and curcumin in patients with ongoing inflammatory activity appears to be effective as measured by CDAI and Harvey–Bradshaw Index.

## 1 Introduction

Crohn's disease (CD) is a complex chronic inflammatory disease of the gastrointestinal tract, the most accepted mechanisms involved in the pathogenesis are immunological mediation in genetically susceptible individuals and environmental factors that trigger the onset of disease by affecting the mucosal barrier. The healthy balance of gut microbiota is also altered which stimulates complex immune responses [1]. Several melanocortins seem to have a role in inflammatory bowel disease pathogenesis. Melanocytes modulate inflammatory processes [2]. This may lead to the development of new therapeutic avenues for the treatment of inflammatory bowel diseases.

CD is treated with lifelong immunosuppressive medication. Purine analogues such as azathioprine (AZT) and 6‐mercaptopurine (6‐MP) have been used to maintain remission in CD, but the effectiveness, tolerability, and safety of these agents remains controversial. The maintenance of remission is accomplished with long‐term AZT treatment after an initial round of steroids to induce disease control [3]. Around 45% of patients who take AZT over the long‐term remain in remission. For disease that does not respond to AZT, anti‐TNF therapy such as infliximab and adalimumab is increasingly employed. Such treatment options are not only expensive but can also reactivate latent tuberculosis in approximately 10% patients [4].

The potent anti‐inflammatory capabilities of naturally occurring compounds like artemisinins and curcumin have recently attracted renewed interest. Both these agents have demonstrated encouraging anti‐inflammatory activity in a small number of patients as well as in animal models. Artemisinin was isolated from the *Artemisia* (genus) plant in 1971 and is a medicinal natural product commonly used in the treatment of malaria [5]. Recent studies have shown that artemisinin derivatives also exert antitumor effects [6, 7, 8, 9, 10], which have attracted attention for use as anticancer drugs. Due to its anti‐inflammatory properties, artesunate (ART), a semisynthetic derivative of artemisinin, has been used in the treatment of several inflammatory diseases. It has also been reported that ART can inhibit the activation of the Toll‐like receptor 4 (TLR4)‐nuclear factor (NF)‐κB pathway [11]. In a mouse model of Dextran sodium sulfate (DSS)‐induced colitis, curcumin has been demonstrated to have protective effects through regulation of TNF‐alpha release [12]. When compared to placebo, it has been demonstrated to be effective in studies involving patients with active and dormant ulcerative colitis [13]. Curcumin, as a natural agent with a low price, has less adverse reactions and high safety drug use. Curcumin significantly reduces the activity of myeloperoxidase. This results in reduction of oxidative stress and cytokine cascade [14]. Curcumin has also been shown to inhibit melanogenesis in human melanocytes [15]. On the other hand, ART has been shown to suppress Tumor Necrosis Factor alpha (TNF‐α) expression and T‐helper (Th)1/Th17 responses in a Trinitrobenzene Sulfonic acid (TNBS) colitis model [16, 17].

We aimed to study the safety, tolerability, and efficacy of ART and Curcumin in patients with CD, who have ongoing clinical and biochemical evidence of disease activity despite treatment with AZT.

## 2 Methods

### 2.1 Study Site

This pilot study was conducted at the Department of Gastroenterology, Sanjay Gandhi Postgraduate Institute of Medical Sciences, which is a university teaching tertiary referral center in northern India.

### 2.2 Trial Design

This is a single‐center phase IIa, randomized, double‐blind, placebo‐controlled factorial design, proof of concept trial. Following recruitment, block randomization was done using concealed allocation. Block randomization in a 1:1:1:1 ratio was carried out in blocks of 10 patients in each group. Concealed allocation of the patients to different groups was undertaken by closed envelop technique. The trial details have previously been published (https://clinicaltrials.gov/study/NCT04713631).

### 2.3 Inclusion Criteria

Patients, aged 18–65 years, with a diagnosis of CD who still had mild to moderate inflammatory activity (CDAI 150–450) despite being treated with an adequate, constant dose of AZT for at least 3 months were included after obtaining informed consent. The patients were treated with glucocorticoids initially to induce remission and then switched to AZT to maintain remission. None of the patients was on steroids at the time of recruitment into the study.

### 2.4 Exclusion Criteria

Children with a diagnosis of CD, pregnant/lactating women, patients who had bowel surgery within the past 3 months, intra‐abdominal abscess, ileostomy, or colostomy, change in dose of 5‐Aminosalicylic acid (ASA) in the past 4 weeks, or use of corticosteroids within the past 4 weeks were excluded. Patients with abnormalities on their ECG were also excluded.

### 2.5 Recruitment

Patients with a diagnosis of CD attending the gastroenterology outpatient clinic at Sanjay Gandhi Postgraduate Institute of Medical Sciences, Lucknow, India from November 2021 to November 2022 were recruited.

### 2.6 Intervention

Patients who were receiving an adequate and constant dose of AZT for 3 months and still continued to have mild to moderate active disease (Crohn's Disease Activity Index [CDAI] > 150) were enrolled. Eighty one patients with CD were screened for recruitment to the study. Forty patients were randomized in a 1:1:1:1 ratio into four groups in a 2 × 2 factorial design to receive ART 200 mg orally daily (4 mg/kg with a maximum of 200 mg) for 2 weeks and/or Curcumin (Cadila Pharmaceuticals, India) 200 mg orally daily for 3 months or placebo. Patients weighing less than 50 kg, received a reduced dose of 150 or 100 mg of ART, ensuring that it did not exceed 4 mg/kg/.

### 2.7 Follow‐Up

The patients continued with a regular dose of AZT regardless of which group they were randomized into, with no change during the study period. Patients were asked to maintain a daily diary of symptoms and adverse events. Scheduled hospital visits with blood and stool tests were undertaken at baseline, Week 1, Month 1, Month 3, and Month 6. Pill count was done during each patient's visit to the hospital to assess drug compliance.

### 2.8 Daily Diary

Data that were recorded daily in the patient diary at home included

1. General well‐being (very well/slightly below par/poor/very poor/terrible)
2. Number of loose (liquid or soft) stools a day
3. Abdominal pain (none/mild/moderate/severe)
4. Abdominal mass
5. Extraintestinal manifestations (arthralgia/uveitis/erythema nodosum/aphthous ulcers/pyoderma gangrenosum/active anal fissure/new fistula/abscess)
6. Adverse events
  - Allergic drug reaction
  - Worsening of the disease more than that expected in the natural course of the disease
    - Rise in CDAI score of > 100 from baseline or HBS of 3 from baseline after 4 weeks from study commencement
    - New onset or aggravation of extraintestinal manifestations, after 4 weeks from enrollment.

The diary was checked at each visit. If the patient had entered anything suggestive of a mass or extraintestinal manifestation, it was checked by the physician.

### 2.9 Sample Collection

Blood and stool samples were taken at baseline, after 1 week of medication, after 1 month, after 3 months and after 6 months. Each blood sample was analyzed for hemoglobin, hematocrit, platelet count, white blood cells, aspartate aminotransferase, alanine aminotransferase, alkaline phosphatase, bilirubin, creatinine, C‐reactive protein, erythrocyte sedimentation rate. Fecal samples were taken at the same time points to measure calprotectin.

### 2.10 Statistical Analysis

Categorical and continuous data were presented as percentages, medians, and ranges. Chi‐squared or Fischer's exact tests were used to compare the categorical variables. Kruskal–Wallis H test was conducted to assess differences in scores of CDAI, Harvey–Bradshaw Index, Fecal Calprotectin, and blood test results across all four treatment groups. Post hoc pairwise comparisons were made using the Dwass‐Steel‐Critchlow‐Fligner (DSCF) test. The change from baseline at various time points was calculated for CDAI, Harvey–Bradshaw Index, and Fecal Calprotectin. The Kruskal–Wallis test was used to check differences in change between all four treatment groups. Within each treatment group, the Freidman test was used to check for differences in CDAI, Harvey–Bradshaw Index, and Fecal Calprotectin scores at all time points from Baseline to 6 months. Post hoc pairwise comparisons were conducted using the DSCF test. All analyses were performed using SAS 9.4 with a significance level of *p* < 0.05. More robust statistical analyses, such as linear mixed‐effects models, are not feasible. While such models might technically fit the data, the small sample size of 40 individuals across the four treatment groups would undermine the precision and interpretability of the results, making it difficult to draw meaningful conclusions. Additionally, overfitting is a concern in small datasets, as the models may capture noise rather than true effects, leading to results that are specific to this dataset but not generalizable. For these reasons, we opted for the Friedman test and DSCF pairwise comparisons, which are well‐suited for small, repeated‐measures datasets and provide valid and interpretable insights.

## 3 Results

### 3.1 Patient Flow

**FIGURE 1:**
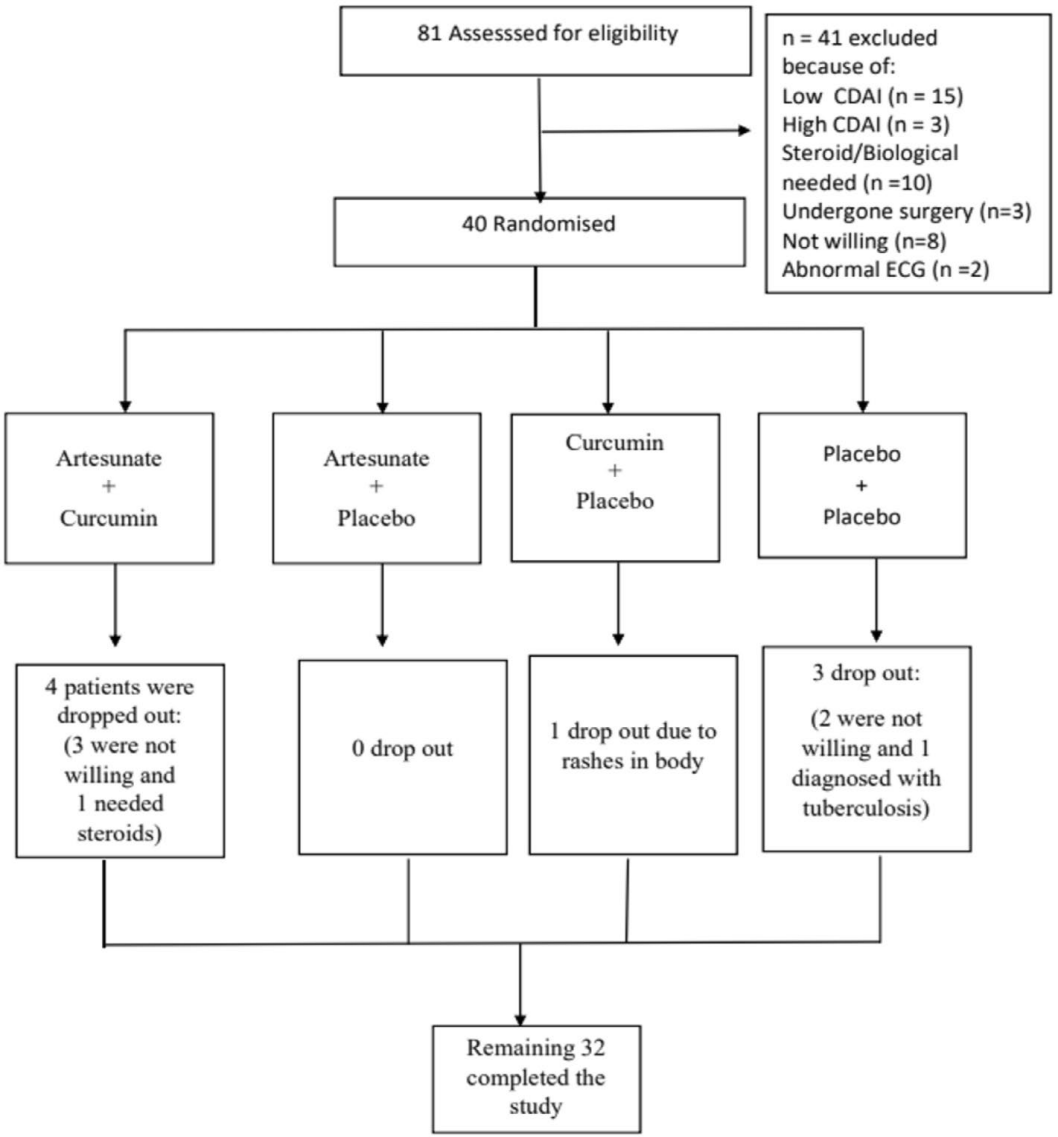
Flow chart showing the randomization and progress of patients in the four groups.

Figure 1 summarizes patient flow. Eighty‐one patients were screened. 40/81 patients who fulfilled the inclusion criteria were randomized (10 patients in each group). As there was no guidance from previous studies, an exact sample size could not be calculated. An arbitrary number of 40 patients was, therefore, chosen. Eight patients could not continue the study, five patients dropped out because of unwillingness to continue with the study, one needed steroids, one patient was diagnosed with tuberculosis and another patient developed a rash on his body (Figure 1). Recruitment ended after the planned numbers were randomized.

Out of the 40 patients in the sample, 30 were male (75.0%) and 21 were from the upper middle socioeconomic class (52.5%). Twenty‐five out of 40 (62.5%) patients followed a vegetarian diet, and 34 patients (85.0%, *p* = 0.03) did not smoke or consume alcohol or chew tobacco. Nineteen patients (47.5%) had a previous history of a blood transfusion and 21 patients had received antitubercular therapy (ATT) (52.5%). Twenty‐one patients reported weight loss (52.5%), 24 loss of appetite (60.0%), and 27(67.5%) patients reported suffering from fatigue. There were no significant differences in the distribution of these characteristics across all four treatment groups, except for addiction to tobacco (*p* = 0.0376). The ART + Curcumin (20.0%) and Placebo + Placebo (40.0%) groups had patients with a history of smoking, chewing tobacco, and alcohol consumption (Table 1).

**TABLE 1:** Distribution of patients by treatment group.

	Treatment group										Fisher's exact Test *p* value
	Artesunate + Curcumin		Artesunate + Placebo		Curcumin + Placebo		Placebo + Placebo		Total		
	*N*	Col percent	*N*	Col percent	*N*	Col percent	*N*	Col percent	*N*	Percent	
Gender											
Female	3	30.0	1	10.0	5	50.0	1	10.0	10	25.0	0.1972
Male	7	70.0	9	90.0	5	50.0	9	90.0	30	75.0	
Socioeconomic status											
Lower	1	10.0							1	2.5	0.9923
Lower middle	2	20.0	2	20.0	2	20.0	3	30.0	9	22.5	
Upper	1	10.0	1	10.0			1	10.0	3	7.5	
Upper lower	2	20.0	1	10.0	2	20.0	1	10.0	6	15.0	
Upper middle	4	40.0	6	60.0	6	60.0	5	50.0	21	52.5	
Diet											
Nonvegetarian	2	20.0	4	40.0	4	40.0	5	50.0	15	37.5	0.6622
Vegetarian	8	80.0	6	60.0	6	60.0	5	50.0	25	62.5	
Addiction to smoking/alcohol/tobacco											
No	8	80.0	10	100.0	10	100.0	6	60.0	34	85.0	0.0376*
Yes	2	20.0					4	40.0	6	15.0	
Blood transfusion in past											
No	6	60.0	6	60.0	6	60.0	3	30.0	21	52.5	0.5380
Yes	4	40.0	4	40.0	4	40.0	7	70.0	19	47.5	
ATT in the past											
No	6	60.0	3	30.0	5	50.0	5	50.0	19	47.5	0.6843
Yes	4	40.0	7	70.0	5	50.0	5	50.0	21	52.5	
Weight loss											
No	4	40.0	3	30.0	5	50.0	7	70.0	19	47.5	0.4026
Yes	6	60.0	7	70.0	5	50.0	3	30.0	21	52.5	
Loss of appetite											
No	4	40.0	1	10.0	6	60.0	5	50.0	16	40.0	0.1273
Yes	6	60.0	9	90.0	4	40.0	5	50.0	24	60.0	
Fatigue											
No	5	50.0	1	10.0	4	40.0	3	30.0	13	32.5	0.3414
Yes	5	50.0	9	90.0	6	60.0	7	70.0	27	67.5	

* *p* ≤ 0.05.

Hematological and biochemical parameters did not show any evidence of adverse drug effects to either ART or curcumin. However, the creatinine value did differ from baseline and following treatment (*p* = 0.0187, Kruskal–Wallis H test) (Table 2). Post hoc DSCF test revealed a group difference in the creatinine level at baseline among curcumin + placebo and placebo + placebo (*p* = 0.0498). However, all the values remained within the upper limits of normal.

**TABLE 2:** Median (min—max) scores across treatment groups.

Test	Visit	Artesunate + Curcumin	Artesunate + Placebo	Curcumin + Placebo	Placebo + Placebo	Kruskal–Wallis test *p* value
CDAI	Baseline	190.0 (163.0–430.0)	196.5 (150.0–440.0)	206.0 (150.0–440.0)	184.0 (155.0–276.0)	0.8984
	1 week	166.0 (110.0–275.0)	138.5 (101.0–279.0)	164.5 (7.0–350.0)	176.0 (57.0–250.0)	0.8108
	1 month	139.5 (10.0–289.0)	120.5 (69.0–414.0)	116.0 (33.0–245.0)	116.0 (19.0–250.0)	0.8133
	3 months	70.5 (37.0–118.0)	128.5 (50.0–198.0)	144.0 (37.0–220.0)	125.0 (0.0–234.0)	0.2871
	6 months	65.0 (40.0–157.0)	55.5 (1.0–425.0)	138.0 (53.0–193.0)	110.0 (1.0–411.0)	0.1462
Harvey–Bradshaw	Baseline	3.0 (2.0–8.0)	4.0 (2.0–8.0)	3.0 (2.0–9.0)	3.0 (2.0–5.0)	0.9159
	1 week	3.0 (2.0–4.0)	3.0 (0.0–5.0)	2.5 (0.0–8.0)	3.0 (0.0–4.0)	0.7526
	1 month	2.5 (0.0–4.0)	2.0 (0.0–6.0)	1.0 (0.0–4.0)	1.0 (0.0–4.0)	0.8804
	3 months	0.0 (0.0–3.0)	1.0 (0.0–5.0)	1.0 (0.0–4.0)	2.0 (0.0–5.0)	0.1015
	6 months	0.0 (0.0–1.0)	0.0 (0.0–5.0)	3.0 (0.0–6.0)	2.0 (0.0–14.0)	0.0447*
Fecal calprotectin	Baseline	127.0 (27.0–893.1)	108.8 (39.7–3000.0)	128.5 (52.0–1872.0)	130.9 (35.0–893.1)	0.7738
	1 week	35.8 (8.6–591.0)	181.0 (47.0–2500.0)	296.3 (5.4–774.2)	53.9 (5.0–893.1)	0.2306
	1 month	53.0 (37.0–355.2)	91.0 (25.0–1110.0)	117.0 (38.0–699.0)	88.9 (52.0–893.1)	0.4935
	3 months	93.5 (5.0–383.0)	108.5 (27.0–496.0)	191.5 (18.0–745.0)	76.0 (19.0–1829.0)	0.8860
	6 months	45.5 (25.0–381.0)	71.0 (26.3–854.2)	339.7 (110.0–548.8)	88.4 (27.0–3000.0)	0.2529
ESR	Baseline	26.5 (9.0–58.0)	32.0 (9.0–91.0)	32.0 (8.0–105.0)	21.0 (5.0–70.0)	0.4174
	1 week	28.0 (8.0–81.0)	31.0 (8.0–78.0)	42.5 (7.0–91.0)	27.0 (10.0–48.0)	0.6944
	1 month	40.0 (6.0–65.0)	30.5 (9.0–50.0)	42.0 (15.0–89.0)	31.0 (8.0–77.0)	0.6658
	3 months	40.0 (20.0–53.0)	27.0 (15.0–74.0)	40.0 (10.0–86.0)	20.0 (7.0–76.0)	0.826
	6 months	42.0 (7.0–82.0)	27.5 (2.0–72.0)	45.0 (8.0–140.0)	19.0 (17.0–80.0)	0.4151
CRP	Baseline	0.3 (0.0–4.7)	0.5 (0.0–11.5)	0.4 (0.0–4.2)	0.2 (0.0–0.8)	0.7919
	1 week	3.0 (0.0–7.0)	2.0 (0.0–12.0)	1.0 (0.0–2.0)	0.0 (0.0–4.0)	0.1246
	1 month	0.9 (0.2–1.8)	0.7 (0.1–4.3)	0.6 (0.1–2.2)	0.3 (0.0–2.8)	0.4681
	3 months	1.2 (0.0–5.6)	1.8 (0.1–11.4)	0.6 (0.1–3.1)	0.2 (0.0–3.1)	0.3136
	6 months	0.3 (0.0–5.6)	0.4 (0.1–3.6)	0.3 (0.1–7.5)	0.4 (0.0–2.5)	0.9282
HGB	Baseline	12.8 (9.1–15.0)	12.7 (9.0–15.0)	12.0 (9.0–14.0)	12.8 (9.0–17.3)	0.4358
	1 week	11.1 (8.0–15.0)	11.3 (8.0–14.9)	11.0 (8.7–15.3)	12.5 (9.7–15.6)	0.5094
	1 month	12.0 (9.9–15.8)	11.6 (8.0–14.7)	10.7 (9.1–15.3)	13.0 (8.7–15.8)	0.3718
	3 months	10.7 (6.9–14.8)	11.9 (8.0–15.0)	10.0 (6.4–12.8)	13.0 (10.0–15.8)	0.155
	6 months	11.5 (8.2–15.8)	11.8 (7.0–15.3)	9.9 (5.4–12.0)	12.0 (7.9–16.4)	0.1355
HCT	Baseline	40.2 (33.1–46.0)	36.0 (28.0–47.1)	34.4 (27.0–44.7)	38.4 (27.0–53.2)	0.1573
	1 week	38.1 (26.2–40.7)	35.7 (26.0–43.8)	34.1 (28.0–48.2)	37.5 (32.0–47.7)	0.6751
	1 month	35.9 (32.1–42.0)	37.9 (30.0–44.8)	33.5 (28.0–47.5)	41.8 (30.0–47.9)	0.2783
	3 months	35.4 (24.6–44.8)	38.1 (26.0–44.5)	34.7 (25.0–40.1)	42.0 (33.6–46.5)	0.2022
	6 months	37.0 (27.0–47.0)	38.5 (25.0–48.0)	31.0 (20.0–42.0)	38.0 (32.0–50.0)	0.0811
PLT	Baseline	208.5 (130.0–377.0)	279.0 (112.0–782.0)	207.0 (110.0–519.0)	223.0 (110.0–330.0)	0.938
	1 week	224.0 (130.0–290.0)	278.5 (110.0–571.0)	258.0 (133.0–409.0)	263.0 (163.0–310.0)	0.5003
	1 month	218.5 (173.0–347.0)	255.0 (105.0–428.0)	214.0 (98.0–420.0)	232.0 (155.0–432.0)	0.9875
	3 months	235.0 (140.0–468.0)	252.0 (82.0–725.0)	312.0 (98.0–447.0)	262.0 (155.0–432.0)	0.9963
	6 months	283.5 (140.0–470.0)	284.0 (2.0–735.0)	280.0 (110.0–615.0)	326.0 (168.0–471.0)	0.8296
TLC	Baseline	6.8 (4.6–11.2)	6.9 (4.0–10.0)	5.0 (3.7–6.8)	7.1 (4.7–13.6)	0.0506
	1 week	6.3 (3.7–10.0)	4.9 (1.1–7.5)	5.8 (3.1–8.6)	5.1 (4.0–7.3)	0.4967
	1 month	7.1 (1.9–8.4)	5.0 (3.5–7.4)	5.7 (4.7–7.1)	5.7 (4.3–8.6)	0.3223
	3 months	5.8 (2.4–10.3)	5.7 (3.0–11.0)	5.0 (3.9–7.2)	5.3 (4.0–7.3)	0.8271
	6 months	6.5 (2.0–7.0)	6.0 (3.0–14.0)	4.0 (4.0–9.0)	7.0 (6.0–12.0)	0.3548
SGPT	Baseline	18.5 (9.0–62.0)	17.5 (8.0–36.0)	17.5 (7.0–37.0)	22.5 (10.0–75.0)	0.4083
	1 week	27.0 (11.0–50.5)	18.0 (7.0–30.0)	22.0 (8.0–70.0)	26.0 (12.0–47.0)	0.3954
	1 month	20.0 (8.0–36.0)	18.0 (11.0–28.0)	15.0 (8.0–41.0)	21.0 (13.0–60.0)	0.6671
	3 months	25.0 (18.0–56.0)	18.0 (10.0–30.0)	17.0 (10.0–96.0)	21.0 (18.0–62.0)	0.1069
	6 months	22.0 (17.0–51.0)	20.5 (10.0–40.0)	21.0 (8.0–38.0)	23.0 (12.0–87.0)	0.7015
SGOT	Baseline	22.0 (12.0–37.0)	23.0 (12.0–41.0)	23.0 (15.0–57.0)	26.0 (9.0–61.0)	0.8647
	1 week	29.0 (20.0–59.5)	25.5 (12.0–35.5)	23.0 (15.0–147.0)	27.0 (21.0–67.0)	0.5237
	1 month	21.0 (12.0–36.0)	25.0 (13.0–38.0)	23.0 (16.0–60.0)	26.0 (12.0–53.0)	0.607
	3 months	21.0 (12.0–45.0)	22.5 (14.0–34.0)	22.0 (14.0–124.0)	25.0 (16.0–43.0)	0.5977
	6 months	22.5 (10.0–36.0)	26.5 (18.0–42.0)	23.0 (17.0–72.0)	31.0 (12.0–56.0)	0.5413
ALP	Baseline	86.0 (51.0–171.0)	84.5 (64.0–109.0)	70.5 (54.0–176.0)	80.0 (51.0–114.0)	0.4794
	1 week	107.0 (52.0–167.0)	80.0 (62.0–256.0)	75.0 (53.0–334.0)	76.3 (49.0–112.0)	0.8663
	1 month	92.5 (52.0–137.0)	85.5 (70.0–124.0)	70.0 (53.0–310.0)	75.0 (51.0–108.0)	0.4731
	3 months	64.5 (43.0–118.0)	80.0 (60.0–125.0)	71.0 (53.0–290.0)	85.0 (32.0–184.0)	0.6867
	6 months	65.0 (41.0–131.0)	78.5 (67.0–125.0)	65.0 (45.0–210.0)	77.0 (32.0–110.0)	0.3391
Bilirubin total	Baseline	0.9 (0.3–1.1)	0.7 (0.2–1.0)	0.5 (0.3–1.5)	1.0 (0.5–1.6)	0.1729
	1 week	0.6 (0.3–0.9)	0.5 (0.3–1.5)	0.6 (0.3–1.0)	0.9 (0.4–1.6)	0.1647
	1 month	0.8 (0.3–1.0)	0.8 (0.4–2.5)	0.6 (0.4–1.5)	1.1 (0.4–1.8)	0.7264
	3 months	0.6 (0.3–0.7)	0.8 (0.4–1.3)	0.7 (0.3–1.7)	0.5 (0.3–1.2)	0.2189
	6 months	0.6 (0.1–0.7)	0.7 (0.4–2.7)	0.7 (0.3–1.6)	0.8 (0.2–1.2)	0.4901
Bilirubin direct	Baseline	0.3 (0.0–1.0)	0.2 (0.1–0.6)	0.3 (0.1–0.6)	0.4 (0.2–0.6)	0.1282
	1 week	0.2 (0.1–0.6)	0.2 (0.1–0.4)	0.3 (0.1–0.5)	0.3 (0.2–0.7)	0.3572
	1 month	0.3 (0.1–0.3)	0.3 (0.2–0.7)	0.3 (0.2–0.7)	0.5 (0.2–0.7)	0.3712
	3 months	0.2 (0.1–0.3)	0.3 (0.2–0.7)	0.3 (0.2–0.6)	0.3 (0.1–0.5)	0.2936
	6 months	0.3 (0.1–0.3)	0.3 (0.2–0.5)	0.3 (0.2–0.8)	0.5 (0.1–0.8)	0.4583
Creatinine	Baseline	1.0 (0.7–1.1)	0.9 (0.7–1.0)	0.7 (0.6–1.1)	1.0 (0.8–1.2)	0.0187*
	1 week	1.0 (0.2–1.2)	0.9 (0.1–1.0)	0.7 (0.6–1.1)	1.0 (0.8–1.2)	0.4460
	1 month	0.8 (0.2–1.1)	0.9 (0.6–1.1)	0.7 (0.6–1.1)	0.9 (0.8–1.2)	0.2322
	3 months	0.9 (0.7–1.2)	0.8 (0.7–1.0)	0.7 (0.6–1.1)	1.0 (0.8–1.1)	0.052
	6 months	1.0 (0.9–1.2)	0.8 (0.7–1.1)	0.8 (0.6–1.1)	0.9 (0.6–1.3)	0.0976

* *p* ≤ 0.05.

The overall mean score at baseline for CDAI was 217.7 (SD: 79.2), Harvey–Bradshaw index was 3.8 (SD: 1.8) and Fecal Calprotectin was 368.9 (SD: 589.5). At 6 months, the overall mean score for CDAI was 125.5 (SD: 105.1), Harvey–Bradshaw index was 1.9 (SD: 3.1) and Fecal Calprotectin was 336.8 (SD: 589.3). Kruskal–Wallis test showed that the Harvey–Bradshaw Index statistically differed across the treatment groups at 6 months (*p* = 0.047), however, there was no significant group differences in the post hoc pairwise analysis. The change from baseline scores was not statistically different across all groups for all time points.

Friedman test for repeated measures showed group differences in scores for CDAI, Harvey–Bradshaw Index, and Fecal Calprotectin at different time points among ART + Curcumin, ART + Placebo, and Curcumin + Placebo groups. Post hoc pairwise comparisons using DSCF test showed that differences in CDAI from baseline to 6 months were statistically significant in ART + Curcumin (*p* = 0.0098) and Curcumin + Placebo (*p* = 0.0431) groups. Similarly, statistically significant differences were observed between Baseline and 6 months for the Harvey–Bradshaw Index in the ART + Curcumin (*p* = 0.0070) and Curcumin + Placebo (*p* = 0.0138) groups. There were no significant differences in the post hoc analysis for CDAI in the ART + Placebo group, and the Harvey–Bradshaw Index in the Curcumin + Placebo group (Table 3). There was no significant difference either within groups at various time points or across the four groups.

**TABLE 3:** Post hoc DSCF *p* values for pairwise comparisons between different time points.

Outcome	Group	vs. time point	Baseline	1 week	1 month	3 months
CDAI	Artesunate + Curcumin	1 week	0.9040	—	—	—
		1 month	0.3467	0.8555	—	—
		3 months	0.0099*	0.0519	0.3964	—
		6 months	0.0098*	0.0343*	0.5993	0.9891
	Curcumin + Placebo	1 week	0.7251	—	—	—
		1 month	0.0544	0.5668	—	—
		3 months	0.0836	0.8028	0.9643	—
		6 months	0.0431*	0.9093	0.6474	1.0000
Harvey–Bradshaw Index	Artesunate + Curcumin	1 week	1.0000	—	—	—
		1 month	0.5761	0.5652	—	—
		3 months	0.0316*	0.0438*	0.4837	—
		6 months	0.0070*	0.0154*	0.2815	0.9999
	Artesunate + Placebo	1 week	0.8349	—	—	—
		1 month	0.1785	0.6904	—	—
		3 months	0.0627	0.4131	0.9871	—
		6 months	0.0138*	0.0654	0.2502	0.3502

* *p* ≤ 0.05.

## 4 Discussion

This is the first randomized, double blind controlled pilot trial of ART and curcumin treatment in patients with ongoing inflammatory activity despite treatment with adequate dosage of AZT. Once the acute episode is controlled, patients with CD are maintained in remission using a number of agents including AZT, 6 MP, 5‐ASA compounds, or budesonide. In the western world, there is increasing use of biologics to maintain remission. Despite these strategies, a number of patients continue to suffer from active disease and raised inflammatory markers. Also, the use of biologics as remission maintaining agents may not always be affordable in the developing world.

**FIGURE 2:**
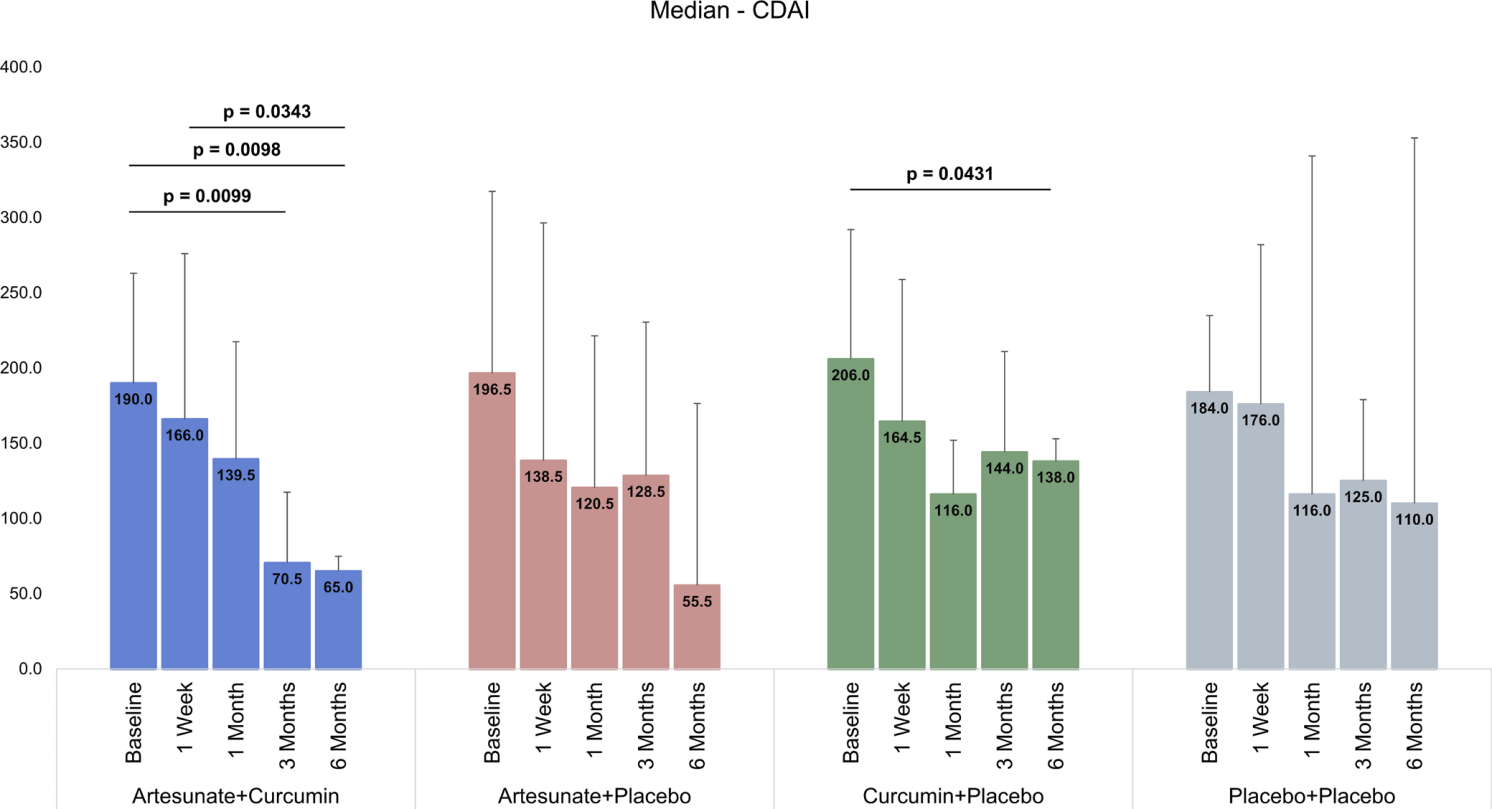
Bar chart showing the effect of four groups of treatment on CDAI.

This study has shown that a combination of ART and curcumin treatment given orally significantly improved the Harvey–Bradshaw index among the four randomized groups and also showed significant improvement in inflammatory markers at different time points from 1 week to 6 months posttreatment. ART and placebo also showed a significant improvement in Harvey–Bradshaw Index at 6 months compared to baseline. Artemisinin, a chemical from a traditional Chinese herbal medicine *Artemisia annua* L., and its derivatives exhibit anti‐inflammatory and immunomodulatory effects in the treatment of systemic lupus erythematosus [18] and rheumatoid arthritis [19] with minimal side effects. ART has excellent water solubility, high stability, and oral bioavailability. ART exerts its pharmacological effects mainly by inhibiting the production of inflammatory factors, reactive oxygen species, autoantibodies, and the migration of cells to reduce damage to tissues or organs [20]. TNF alpha and interleukins (ILs), proinflammatory cytokines, play a significant role in the pathogenesis of inflammatory bowel diseases [21, 22]. In experimental animal models, it has been shown that ART reduces the release of proinflammatory cytokines, TNF alpha, and IL‐6, which helps in controlling the inflammation [23]. It is likely that the anti‐inflammatory effect seen in our study is mediated through its action on proinflammatory cytokines. However, as we did not measure the level of cytokines, it remains to be studied and established in future studies. It is noteworthy that the effect of ART alone was still noticeable at 6 months posttreatment (*p* = 0.0138) after an initial response, suggesting that ART continues to exert its anti‐inflammatory effect long after its initial response in terms of reducing inflammation. Although there was no across‐the‐group effect on CDAI, ART in combination with curcumin showed a significant within‐group effect on CDAI (Figure 2). To prolong the anti‐inflammatory effect and maintain remission, we added curcumin to the initial ART administration. Curcumin has been shown to have anti‐inflammatory, antioxidant, and antitumor effects [24]. Curcumin acts by decreasing the level of proinflammatory mediators [25, 26].

**FIGURE 3:**
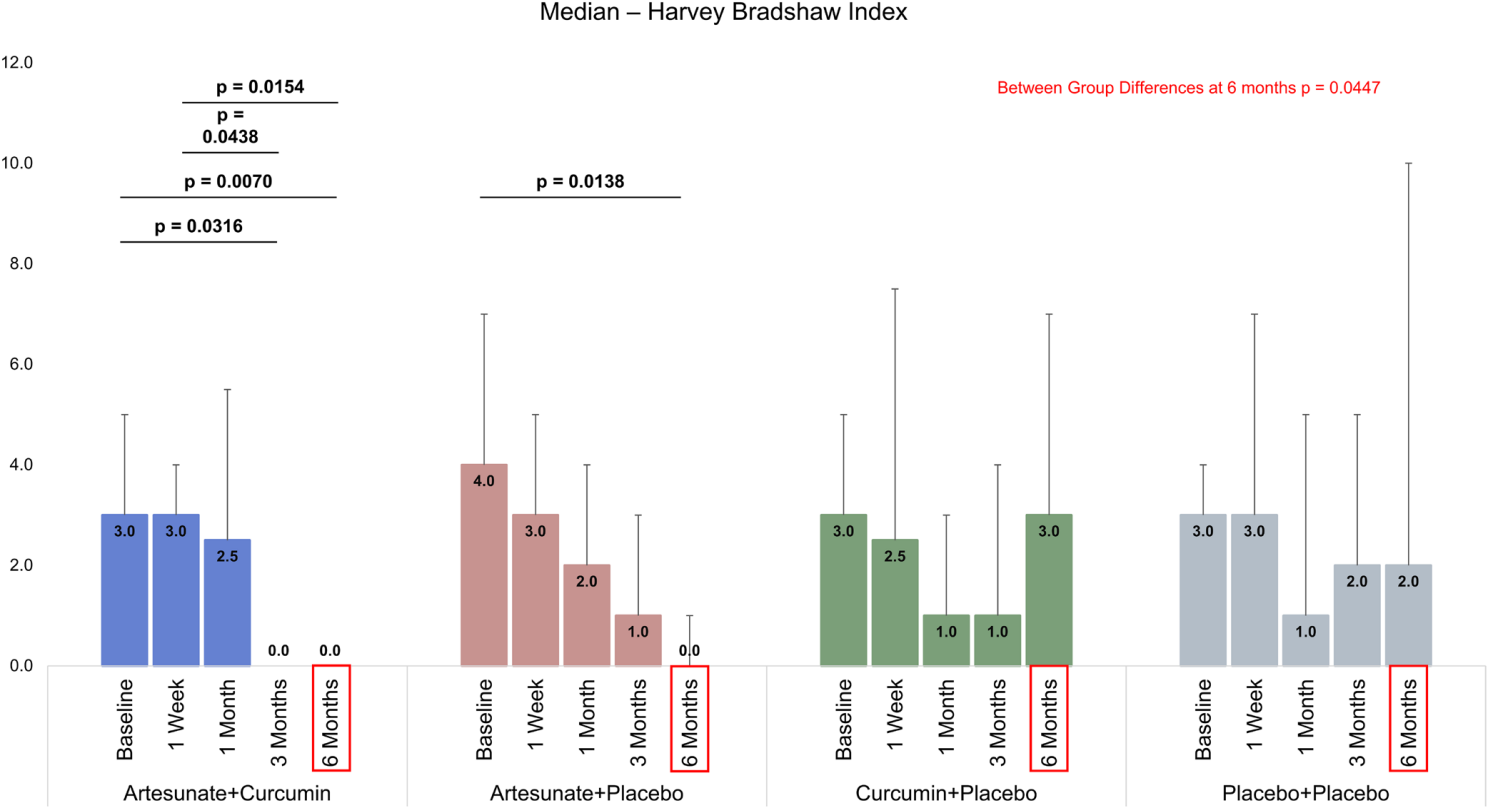
Bar chart showing the effect, within and across groups, of the four different treatment groups on Harvey–Bradshaw Index.

**FIGURE 4:**
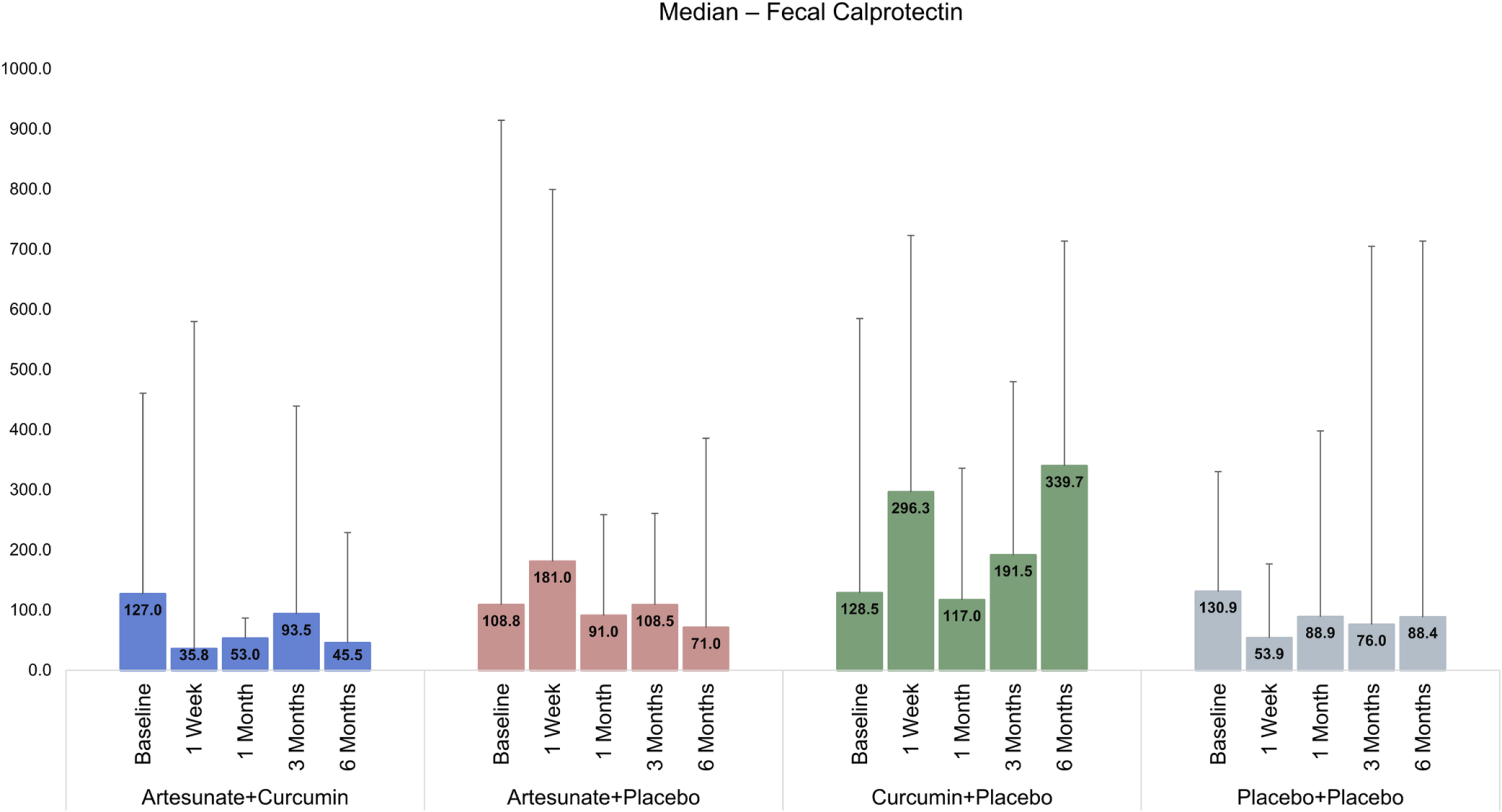
Bar chart showing the effect of the four different treatment groups on Fecal Calprotectin.

Curcumin is a safe and effective adjuvant agent in the treatment of IBD [27, 28, 29]. In patients with IBD, curcumin has a beneficial effect on clinical symptoms, endoscopic relief, and reduction of oxidative stress or inflammatory markers. However, due to the lack of unified standards for curcumin administration form, administration method, dosage, and model selection indices, as well as the limited bioavailability of curcumin, there is still insufficient clinical evidence to prove that curcumin is a therapeutic agent for IBD. Some studies have suggested that oral curcumin was no better than placebo in alleviating clinical symptoms of ulcerative colitis [30]. Curcumin is used in combination with natural ingredients such as emu oil, tetramethylpyrazine, resveratrol, and vitamin D to enhance its anti‐inflammatory effects [31] Researchers now generally agree that curcumin should be used as adjuvant therapy, and when mesalazine is used in the treatment of ulcerative colitis, adding an appropriate amount of curcumin can improve the therapeutic effect of other drugs [32, 33]. Combination therapy is an effective method to improve pharmacokinetics and the anti‐inflammatory effect of curcumin. Combined with piperine, curcumin can synergistically improve anti‐inflammatory and antioxidant activity [34]. It is conceivable that when used in combination with ART, it produces a similarly enhanced effect, although the overlap of curcumin and ART in our study was only for 2 weeks and produced a significant effect in terms of the Harvey–Bradshaw Index, which provides a useful parameter to measure the clinical severity of CD. Curcumin alone, although not as effective as when combined with ART in this study (Figure 3), also produced within‐group significant difference in CDAI at 6 months when compared to baseline (Figure 2). Similarly, fecal calprotectin did not show any significant differences between groups (Figure 4) at all time points. This is likely due to the small sample size. It is hoped that a larger study with bigger numbers will show a significant difference.

We also measured CRP and fecal calprotectin levels at different time points in the four treatment groups. There was no significant difference in CRP levels at different time points either between groups or within groups. Similarly, there was a trend toward improvement in fecal calprotectin but it did not reach statistical significance. This may simply be due to a small sample size. A larger study is now planned to see whether these parameters become significant not only within the group but between groups as well.

Our study has a number of limitations. First, the sample size is small, which makes it difficult to draw firm conclusions. As there is no guidance from previous studies, we were unable to adequately power the study. Second, the follow‐up period of 6 months for a chronic condition such as CD is short. Third, we did not use endoscopy as one of the endpoints. This would be an important consideration in an adequately powered larger study. Also, it is a single‐center study, and it could be argued that the patient population is different and the response is not generalizable.

In conclusion, this pilot study has shown that a combination of ART and curcumin is safe and when used in patients who continue to show inflammatory activity despite treatment with adequate doses of AZT exerts a significant effect as measured by the Harvey–Bradshaw Index. It has also shown that the combination of ART and curcumin has within the group effects at different time points on CDAI and Harvey–Bradshaw Index. A larger study is warranted to establish the clinical efficacy of this combination for the treatment of patients with CD.

## Ethics Statement

The study was approved by the Institutional Ethics Committee (IEC) of Sanjay Gandhi Postgraduate Institute of Medical Sciences, Lucknow, India (IEC Code: 2020‐37‐EMP‐114) and was registered in ClinicalTrials.gov (ID: NCT04713631).

## Conflicts of Interest

The authors declare no conflicts of interest.

## References

[1] R. Boyapati , J. Satsangi , and G. T. Ho , “Pathogenesis of Crohn's Disease,” Primate Report 2 (2015): 7–44. 10.12703/P7-44 PMC4447044 26097717

[2] A. G. Gravina , R. Pellagrino , T. Durante , et al., “The Melanocortin System in IBD: Insights Into the Mechanisms and Therapeutic Potentials,” Cells 12, no. 14 (2023): 1851. 37508552 10.3390/cells12141889 PMC10378568

[3] A. G. Fraser , T. R. Orchard , and D. P. Jewell , “The Efficacy of Azathioprine for the Treatment of Inflammatory Bowel Disease: A 30 Year Review,” Gut 50 (2002): 485–489. 11889067 10.1136/gut.50.4.485 PMC1773162

[4] V. Midha , R. Mahajan , V. Mehta , et al., “Efficacy and Safety of the Adalimumab Biosimilar Exemptia as Induction Therapy in Moderate‐to‐Severe Ulcerative Colitis,” Intestinal Research 16 (2018): 83–89. 29422802 10.5217/ir.2018.16.1.83 PMC5797276

[5] K. Tsuda , L. Miyamoto , S. Hamano , et al., “Mechanisms of the pH‐ and Oxygen‐Dependent Oxidation Activities of Artesunate,” Biological & Pharmaceutical Bulletin 41 (2018): 555–563. 29607928 10.1248/bpb.b17-00855

[6] T. Efferth , M. Giaisi , A. Merling , P. H. Krammer , and M. Li‐Weber , “Artesunate Induces ROS‐Mediated Apoptosis in Doxorubicin‐Resistant T Leukemia Cells,” PLoS One 2 (2007): e693. 17668070 10.1371/journal.pone.0000693 PMC1933253

[7] E. Ooko , M. E. Saeed , O. Kadioglu , et al., “Artemisinin Derivatives Induce Iron‐Dependent Cell Death (Ferroptosis) in Tumor Cells,” Phytomedicine 22 (2015): 1045–1054. 26407947 10.1016/j.phymed.2015.08.002

[8] R. W. Button , F. Lin , E. Ercolano , et al., “Artesunate Induces Necrotic Cell Death in Schwannoma Cells,” Cell Death & Disease 5 (2014): e1466. 25321473 10.1038/cddis.2014.434 PMC4649524

[9] N. Berdelle , T. Nikolova , S. Quiros , T. Efferth , and B. Kaina , “Artesunate Induces Oxidative DNA Damage, Sustained DNA Double‐Strand Breaks, and the ATM/ATR Damage Response in Cancer Cells,” Molecular Cancer Therapeutics 10 (2011): 2224–2233. 21998290 10.1158/1535-7163.MCT-11-0534

[10] N. Eling , L. Reuter , J. Hazin , A. Hamacher‐Brady , and N. R. Brady , “Identification of Artesunate as a Specific Activator of Ferroptosis in Pancreatic Cancer Cells,” Oncoscience 2 (2015): 517–532. 26097885 10.18632/oncoscience.160 PMC4468338

[11] Y. Cen , C. Liu , X. Li , et al., “Artesunate Ameliorates Severe Acute Pancreatitis (SAP) in Rats by Inhibiting Expression of Pro‐Inflammatory Cytokines and Toll‐Like receptor4,” International Immunopharmacology 38 (2016): 252–260. 27318790 10.1016/j.intimp.2016.06.007

[12] H. M. M. Arafa , R. A. Hemeida , A. I. M. El‐Bahrawy , and F. M. A. Hamada , “Prophylactic Role of Curcumin in Dextran Sulfate Sodium (DSS)‐Induced Ulcerative Colitis Murine Model,” Food and Chemical Toxicology 47 (2009): 1311–1317. 19285535 10.1016/j.fct.2009.03.003

[13] H. Hanai , T. Iida , K. Takeuchi , et al., “Curcumin Maintenance Therapy for Ulcerative Colitis: Randomized, Multicenter, Double‐Blind, Placebo‐Controlled Trial,” Clinical Gastroenterology and Hepatology 4 (2006): 1502–1506. 17101300 10.1016/j.cgh.2006.08.008

[14] L. Camacho‐Berqueno , I. Villegas , J. M. Sanchez‐Calvo , et al., “Curcumin, a Curcumina Longa Constituent, Acts on MABK Pathway Modulating COX2 and iNOS Expression in Chronic Experimental Colitis,” International Immunopharmacology 7 (2007): 333–342. 17276891 10.1016/j.intimp.2006.11.006

[15] T. Cai‐Xia , L. Mao , L. Shan‐Shan , Q. Xiao‐yi , Z. Rong‐Xin , and Z. Yun‐Ying , “Curcumin Inhibits Melanogenesis in Human Melanocytes,” Phytotherapy Research 26 (2012): 174–179. 21584871 10.1002/ptr.3517

[16] Z. Yang , J. Ding , C. Yang , et al., “Immunomodulatory and Anti‐Inflammatory Properties of Artesunate in Experimental Colitis,” Current Medicinal Chemistry 19 (2012): 4541–4551. 22834815 10.2174/092986712803251575

[17] L. Hou and H. Huang , “Immune Suppressive Properties of Artemisinin Family Drugs,” Pharmacology & Therapeutics 166 (2016): 123–127. 27411673 10.1016/j.pharmthera.2016.07.002 PMC5035609

[18] Z. Yang , J. Ding , C. Yang , et al., “Immunomodulatory and Anti‐Inflammatory Properties of Artesunate in Experimental Colitis,” Current Medicinal Chemistry 19 (2012): 4541–4551. 22834815 10.2174/092986712803251575

[19] J. Gu , Y. Xu , D. Hua , and Z. Chen , “Role of Artesunate in Autoimmune Diseases and Signaling Pathways,” Immunotherapy 15 (2023): 1183–1193. 37431601 10.2217/imt-2023-0052

[20] T. Efferth and F. Oesch , “The Immunosuppressive Activity of Artemisinin‐Type Drugs Towards Inflammatory and Autoimmune Diseases,” Medicinal Research Reviews 41 (2021): 3023–3061. 34288018 10.1002/med.21842

[21] B. E. Sands and G. G. Kaplan , “The Role of TNF Alpha in Ulcerative Colitis,” Journal of Clinical Pharmacology 47 (2007): 930–941. 17567930 10.1177/0091270007301623

[22] M. Allocca , F. Furfaro , G. Fiorino , D. Gilardi , S. D'Alessio , and S. Danese , “Can IL‐23 Be a Good Target for Ulcerative Colitis?,” Best Practice & Research. Clinical Gastroenterology 32–33 (2018): 95–102. 10.1016/j.bpg.2018.05.016 30060945

[23] Y.‐X. Chen , X.‐Q. Zhang , C.‐G. Yu , et al., “Artesunate Exerts Protective Effects Against Ulcerative Colitis via Suppressing Toll‐Like Receptor 4 and Its Downstream Nuclear Factor‐κB Signaling Pathways,” Molecular Medicine Reports 20, no. 2 (2019): 1321–1332. 31173225 10.3892/mmr.2019.10345 PMC6625425

[24] M. L. Lestari and G. Indrayanto , “Curcumin,” Profiles of Drug Substances, Excipients, and Related Methodology 39 (2014): 113–204. 24794906 10.1016/B978-0-12-800173-8.00003-9

[25] Y. Panahi , M. S. Hosseini , N. Khalili , et al., “Effects of Curcumin on Serum Cytokine Concentrations in Subjects With Metabolic Syndrome: A Post‐Hoc Analysis of a Randomized Controlled Trial,” Biomedicine & Pharmacotherapy 82 (2016): 578–582. 27470399 10.1016/j.biopha.2016.05.037

[26] F. Alizadeh , M. Javadi , A. A. Karami , F. Gholaminejad , M. Kavianpour , and H. K. Haghighian , “Curcumin Nanomicelle Improves Semen Parameters, Oxidative Stress, Inflammatory Biomarkers, and Reproductive Hormones in Infertile Men: A Randomized Clinical Trial,” Phytotherapy Research 32 (2018): 514–521. 29193350 10.1002/ptr.5998

[27] K. Burge , A. Gunasekaran , J. Eckert , and H. Chaaban , “Curcumin and Intestinal Inflammatory Diseases: Molecular Mechanisms of Protection,” International Journal of Molecular Sciences 20 (2019): 1912. 31003422 10.3390/ijms20081912 PMC6514688

[28] F. Fallahi , S. Borran , M. Ashrafizadeh , et al., “Curcumin and Inflammatory Bowel Diseases: From In Vitro Studies to Clinical Trials,” Molecular Immunology 130 (2021): 20–30. 33348246 10.1016/j.molimm.2020.11.016

[29] F. Cunha Neto , L. T. Marton , S. V. de Marqui , T. A. Lima , and S. M. Barbalho , “Curcuminoids From *Curcuma Longa*: New Adjuvants for the Treatment of Crohn's Disease and Ulcerative Colitis?,” Critical Reviews in Food Science and Nutrition 59, no. 13 (2019): 2136–2143. 29565637 10.1080/10408398.2018.1456403

[30] M. R. Coelho , M. D. Romi , D. Ferreira , C. Zaltman , and M. Soares‐Mota , “The Use of Curcumin as a Complementary Therapy in Ulcerative Colitis: A Systematic Review of Randomized Controlled Clinical Trials,” Nutrients 12 (2020): 2296. 32751776 10.3390/nu12082296 PMC7468803

[31] M. S. Hosseini‐Zare , M. Sarhadi , M. Zarei , R. Thilagavathi , and C. Selvam , “Synergistic Effects of Curcumin and Its Analogs With Other Bioactive Compounds: A Comprehensive Review,” European Journal of Medicinal Chemistry 210 (2021): 113072. 33310285 10.1016/j.ejmech.2020.113072

[32] T. Zheng , X. Wang , Z. Chen , A. He , Z. Zheng , and G. Liu , “Efficacy of Adjuvant Curcumin Therapy in Ulcerative Colitis: A Meta‐Analysis of Randomized Controlled Trials,” Journal of Gastroenterology and Hepatology 35 (2020): 722–729. 31696975 10.1111/jgh.14911

[33] S. Kumar , V. Ahuja , M. J. Sankar , A. Kumar , and A. C. Moss , “Curcumin for Maintenance of Remission in Ulcerative Colitis,” Cochrane Database of Systematic Reviews 10 (2012): CD008424. 23076948 10.1002/14651858.CD008424.pub2 PMC4001731

[34] V. S. Ipar , A. Dsouza , and P. V. Devarajan , “Enhancing Curcumin Oral Bioavailability Through Nanoformulations,” European Journal of Drug Metabolism and Pharmacokinetics 44 (2019): 459–480. 30771095 10.1007/s13318-019-00545-z

